# Mucormycosis: an unusual masquerader of an endobronchial tumour

**DOI:** 10.1002/rcr2.488

**Published:** 2019-09-30

**Authors:** Venugopal Jaganathan, Vijaya Prakash Madesh, Santhakumar Subramanian, Rajeshwari K. Muthusamy, Sangita S. Mehta

**Affiliations:** ^1^ Department of Pulmonology Kovai Medical Center and Hospital Coimbatore Tamil Nadu India; ^2^ Department of General Medicine Kovai Medical Center and Hospital Coimbatore Tamil Nadu India; ^3^ Department of Pathology Kovai Medical Center and Hospital Coimbatore Tamil Nadu India

**Keywords:** Endobronchial mucormycosis, endobronchial tumour, poorly controlled diabetes

## Abstract

Pulmonary mucormycosis is a life‐threatening invasive fungal infection usually seen in the background of immunosuppression, haematological malignancies, or uncontrolled diabetes. Immunocompetent hosts can also be affected. Isolated endobronchial mucormycosis is rare with only a few cases reported in the literature. Here, we present a case of an endobronchial mass masquerading as a tumour that was later diagnosed as invasive mucormycosis by histopathological examination.

## Introduction

Pulmonary mucormycosis is a serious opportunistic fungal infection that occurs most commonly in immunocompromised but also reported in an immunocompetent host 1. The major risk factors include prolonged immunosuppression, poorly controlled diabetes mellitus, and haematological malignancies 1. Pulmonary lesions mostly tend to present as consolidation or cavitation. Pulmonary mucormycosis presenting as an endobronchial tumour is rare and only a few cases have been reported 2, 3. They are more commonly seen in patients with poorly controlled diabetes 85% 4, 5. Bronchoscopic evaluation and histopathological examination of the lesions are necessary to differentiate it from a neoplasm. Here, we report a rare presentation of pulmonary mucormycosis in a diabetic patient who presented with left lung collapse. Bronchoscopy revealed an endobronchial growth, mimicking a tumour, causing complete occlusion of the left main bronchus. Histopathology confirmed the diagnosis of invasive mucormycosis.

## Case Report

**Figure 1:**
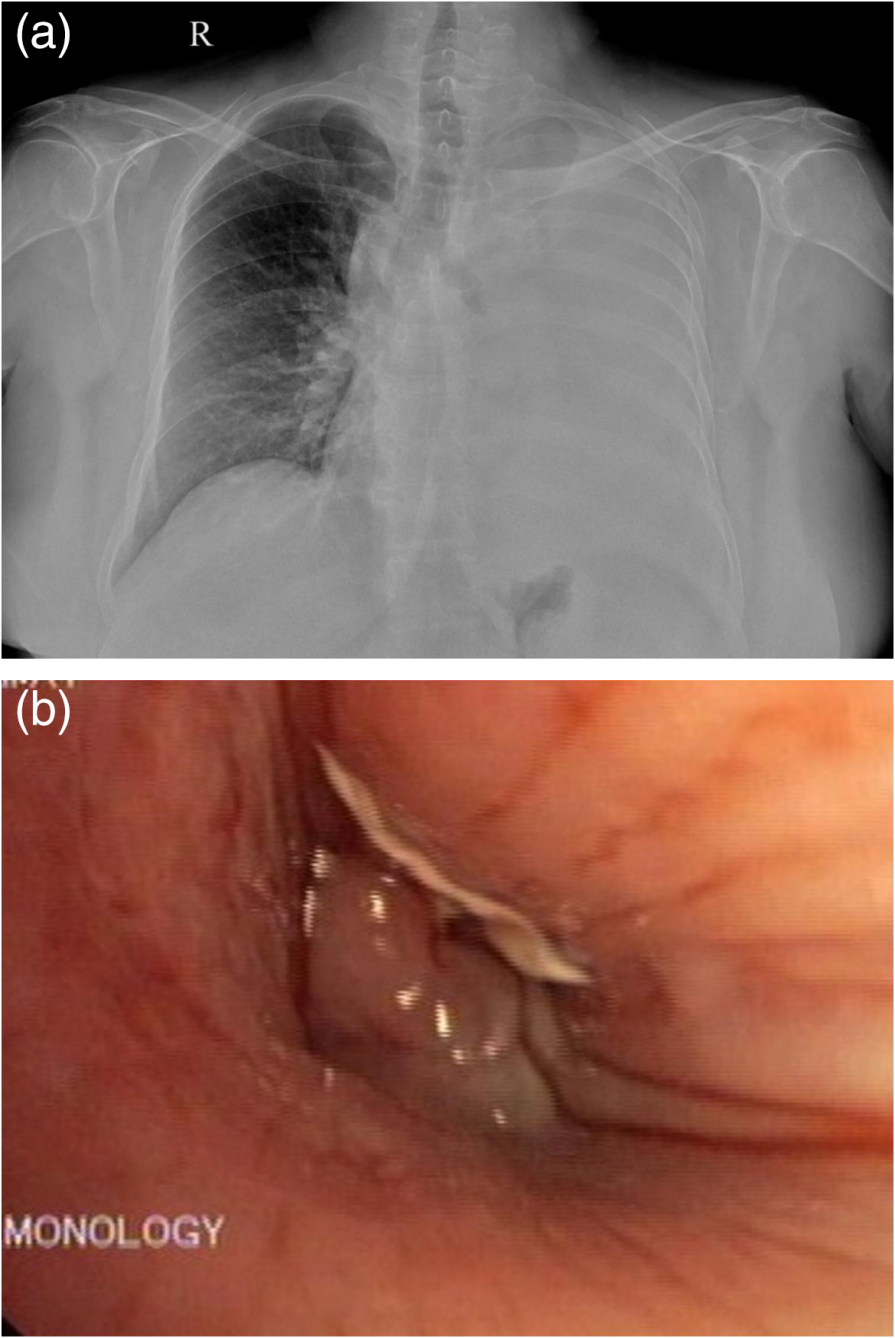
(a) Chest X‐ray showing left lung collapse. (b) Fibre optic bronchoscopy showing an endobronchial mass in the left main bronchus.

**Figure 2:**
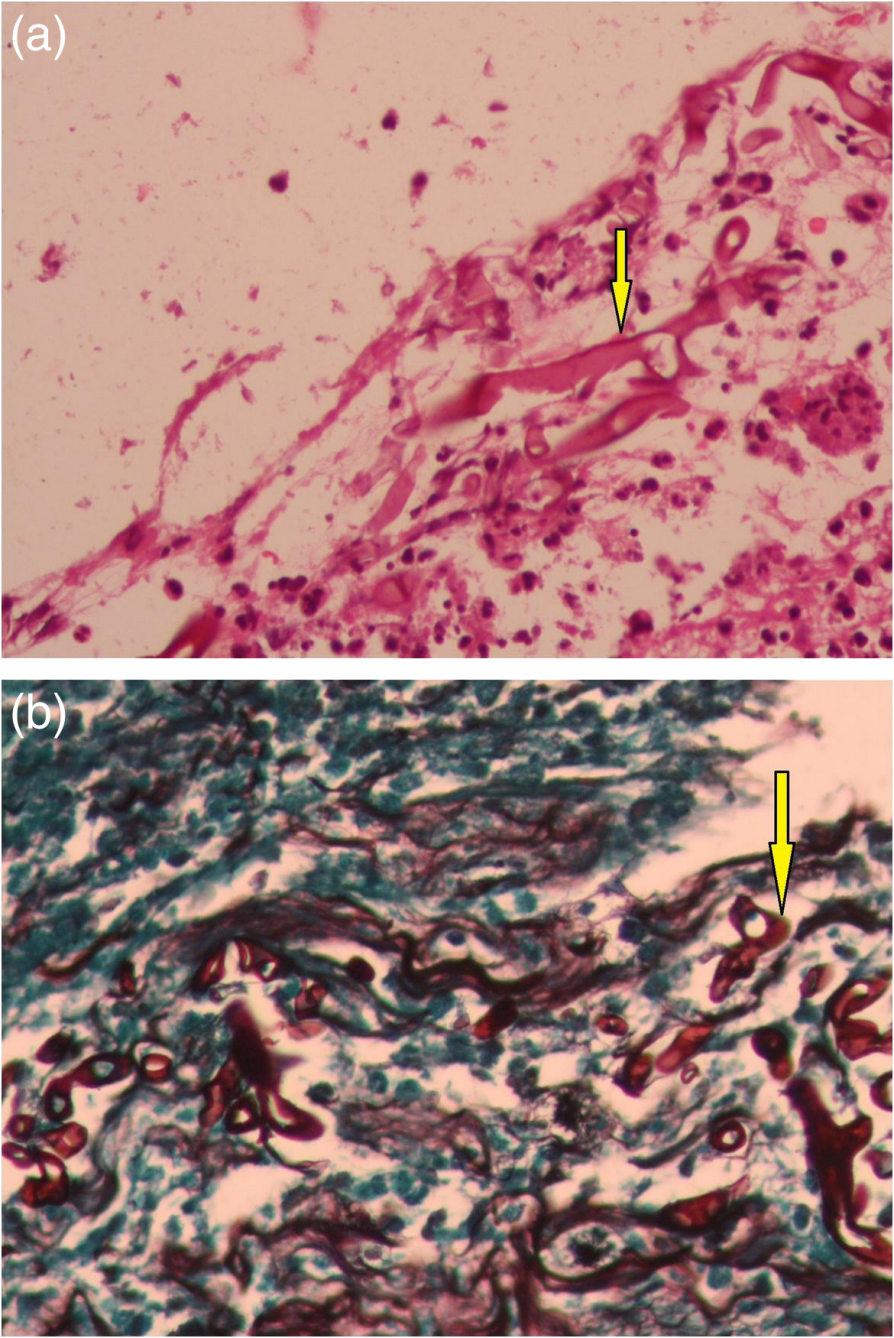
(a) Broad aseptate hyphae within necrotic tissue fragment (yellow arrow). Haematoxylin and eosin: 40×. (b) Gomori methamine silver stain highlighting the aseptate hyphae (yellow arrow) within the vessel wall (magnification: 40×).

A 54‐year‐old woman with poorly controlled diabetes (Glycated hemoglobin [HbA1C] of 10.5%) and grade 4 chronic kidney disease (creatinine of 3.2 mg/dL) presented to the clinic with a history of productive cough and dyspnoea of grade 4 Modified Medical Research Council scale for a period of one week. She had a history of high‐grade squamous intraepithelial lesion on her pap smear a year back for which she has not consulted further. Her physical examination revealed tachypnoea and grossly reduced breath sounds on the left side. Her blood gas analysis on admission showed respiratory alkalosis with partial pressure of Oxygen (PaO_2_) of 64.7 mm Hg and saturation of 94% in room air. Her chest X‐ray revealed a complete homogenous opacity on the left side (Fig. 1a). High‐resolution computed tomography of the thorax showed a complete collapse consolidation of the left lung with abrupt left main bronchial cut off and a mild left pleural effusion. Her cardiac status was normal and the ultrasonogram of the abdomen was normal except for a simple renal cortical cyst in the right kidney. The pleural fluid analysis revealed a sterile effusion, negative for malignancy. Her sputum culture grew methicillin‐resistant *Staphylococcus aureus* for which she was treated with linezolid. Bronchoscopy revealed a fleshy vascular growth completely occluding the left main bronchus (Fig. 1b). Bronchial carcinoid or a bronchogenic carcinoma was suspected. The biopsy was sent for histopathological examination that showed bronchial mucosa and fragments of necrotic tissue with many broad aseptate hyphae invading the stroma and the vessel wall occluding the vascular lumen suggestive for invasive mucormycosis (Fig. 2). Bronchial lavage cytology also revealed aseptate hyphae and was negative for any malignant cells. Histopathology confirmed mucormycosis and so a fungal culture was not performed. The patient and family opted for medical management considering the high risks associated with the surgery. We treated her with oral posaconazole based on her renal functions. She died due to worsening renal failure.

## Discussion

Mucormycosis represents a group of infections caused by the filamentous fungi of the order Mucorales of the subphylum Mucoromycotina (formerly called as Zygomycetes) 5. The term zygomycosis is used synonymously with mucormycosis in the literature. Among the Mucorales, Rhizopus, Absidia, and Mucor are said to be the most common pathogenic organisms 6. Infection primarily occurs by inhalation of its spores and inoculation into the respiratory tract. It can present as localized or disseminated disease. Immunocompromised are more vulnerable to be infected, but cases are also reported in patients with normal immune status 2. The susceptibility of the host is directly associated with phagocytic dysfunction. The common risk factors include poorly controlled diabetes with or without ketoacidosis, immunosuppression, renal failure, neutropenia, long‐term steroid use, or haematological malignancies 4, 6. Desferrioxamine therapy increases the risk of mucormycosis. It chelates with iron forming a siderophore that stimulates fungal growth. Breakthrough zygomycosis has been reported in patients on voriconazole/itraconazole prophylaxis after allogenic haematopoietic stem cell transplant 7. Mucormycosis is a highly invasive and progressive disease with high morbidity and mortality ranging from 25% to 80% 4. It is often underdiagnosed and underreported 2.

Mucormycosis can present as five predominant forms: rhino‐cerebral, pulmonary, cutaneous, gastrointestinal, or disseminated 4. Rhinocerebral and pulmonary are the most common presentations 8. Pulmonary forms are reported to be more common in patients with diabetes (49%), haematological malignancies (28%), and organ transplant and renal failure (11–12%) 9. Their clinical presentation most often resembles bacterial pneumonia with productive or non‐productive cough and dyspnoea. Haemoptysis ranging from trivial to fatal is also reported 10. Common pulmonary manifestations comprise consolidation (66%), cavitation (40%), and less commonly as a solitary pulmonary nodule or mycotic pulmonary artery aneurysms 2, 5.

Pulmonary mucormycosis presenting as an endobronchial lesion is rare. A recent analysis revealed that only 60 cases of mucormycosis involving larger airways have been reported in the literature so far 3. Diabetic patients (85%) have a higher tendency to develop major airway lesions compared to others 1. Steroid therapy (20%) and renal insufficiency (18.3%) comes next in the order. Primary bronchus is the most frequently involved location 3. Most often they present as a solitary mass or infiltrates radiologically. Only 23.2% of the patients are reported to have radiological abnormality suggestive of an endobronchial lesion such as bronchial mass 3. They mimic a malignant lesion bronchoscopically. Bronchoscopic findings are commonly described as an obstructed airway (95%) with a grey‐white mucoid material with surrounding mucosal oedema and necrosis 11. Hyperaemic mucosa can appear in diabetic patients 3. The recovery of Zygomycetes can be difficult in culture. Blood and urine cultures are rarely positive for Zygomycetes, and the rates of successful tissue culture for histopathologically positive smears were reported as 33% and 50% 12. Definite diagnosis is always achieved with biopsy and histopathological examination, which reveals a tissue invasion by aseptate broad right‐angled branching hyphae with a tendency to invade blood vessels 4, 8. Treatment includes antifungals amphotericin‐B or posaconazole, surgery, or combination of both 1, 5. Fungal sepsis followed by respiratory insufficiency and haemoptysis are the common causes of death 4. Patients on medical therapy alone had a mortality of 55% when compared to 27% with surgical treatment with or without medical management 4.

Our patient had both uncontrolled diabetes and renal failure as comorbidities. She presented with an endobronchial mass that was radiologically evident with collapse consolidation of the left lung. Bronchoscopy showed a fleshy vascular growth mimicking a bronchial adenoma or bronchogenic carcinoma. Biopsy confirmed the diagnosis. A high index of suspicion is required for diagnosis.

### Disclosure Statement

Appropriate written informed consent was obtained for publication of this case report and accompanying images.
